# Virus Outbreaks in Chemical and Biological Sensors

**DOI:** 10.3390/s140813592

**Published:** 2014-07-25

**Authors:** Inseong Hwang

**Affiliations:** The Research Institute of Basic Sciences, Seoul National University, Seoul 147-779, Korea; E-Mail: inseong@snu.ac.kr; Tel.: +82-2-888-0762; Fax: +82-2-887-4354

**Keywords:** virus, biosensor, phage display, biomimetic

## Abstract

Filamentous bacteriophages have successfully been used to detect chemical and biological analytes with increased selectivity and sensitivity. The enhancement largely originates not only from the ability of viruses to provide a platform for the surface display of a wide range of biological ligands, but also from the geometric morphologies of the viruses that constitute biomimetic structures with larger surface area-to-volume ratio. This review will appraise the mechanism of multivalent display of the viruses that enables surface modification of virions either by chemical or biological methods. The accommodation of functionalized virions to various materials, including polymers, proteins, metals, nanoparticles, and electrodes for sensor applications will also be discussed.

## 1. Introduction

Over the last two decades, rapid accumulation of genomic and proteomic information has boosted demand for the detection of multiple biomarkers for early and accurate diagnoses as well as advancing the point-of-care of patients. As such, biosensors need to be smaller, smarter, and speedy, while providing quantitative, and sometimes qualitative, information of a chemical or biological element of interest. IUPAC defines a biosensor as “a device that uses specific biochemical reactions mediated by isolated enzymes, immunosystems, tissues, organelles or whole cells to detect chemical compounds usually by electrical, thermal or optical signals [1].” However, current biosensors tend to embrace a variety of mediators beyond those defined recognition receptors to keep up with biomarkers ever increasing in number. Correspondingly, the sensitivity and selectivity of the biological or nonbiological recognition components have become more important factors in sensing devices.

Viruses are biological nanomachines that self-replicate in living cells (hosts) and eventually, by traveling outside the cells, infect other cells. When traveling outside, a complete virus particle, called a virion, recognizes its specific host by receptor-ligand interactions when the infection process begins. To withstand the explosive amplification of virus progenies, living organisms have devised an elaborate immune system regulated by highly-evolved cell-cell and protein-cell interactions of which core again consists of ligand-receptor bindings. Viruses fight back by rapidly and blindly changing their surface ligands to maneuver themselves out of attacks from immune cells. Such evolutionary “playing tag” makes viruses endure surface displaying of a huge diversity of proteins and peptides resulting from frequent mutations.

**Figure 1.**
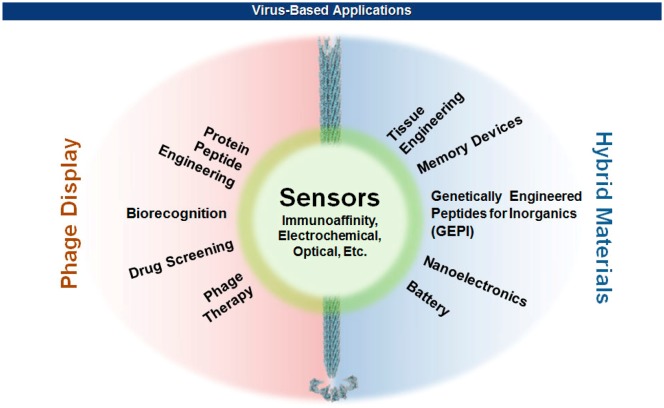
Conventional phage display technology has evolved to reach out virus-based hybrid materials. Both exhibit and share essential characteristics of a transducer interface for the generation of unconventional sensors based on conventional methodologies.

In early 1980s, Smith and coworkers took advantage of the mutagenic tolerance of bacterial viruses by manipulating the viral capsid genes so that proteins or peptides of interest can be displayed on the viral surface [2–4]. Since then, our knowledge about the properties and functions of viral capsids has accumulated as the technologies of genetic engineering and chemical modifications of proteins ripened. On the other hand, recent advances in nanotechnology have branched out to reach viruses that have unique sets of nanostructural dimensions ranging from icosahedral to filamentous shapes. This productive encounter rendered many exciting new opportunities in materials science [5–10], nanotechnology [11,12], and biomedicine [13–15] (Figure 1). This review will focus on the methods of surface displays of bacterial filamentous viruses, and the ease with which various hybrid biomaterials for sensors are made. The effect of multivalency as well as the geometric morphologies of virions on the sensitivity and selectivity of sensors will also be discussed. Interested readers for the detection of pathogenic bacteria using other than filamentous viruses are referred to recent excellent reviews [16–18].

## 2. Filamentous Bacteriophages

Bacteriophages (or simply phages), viruses that parasitize bacteria, can display a reasonable number of combinatorial peptide libraries from which a target-specific binder is selected by a screening process, which is called a phage display technology. Among others, T7 [19], fd [20], and M13 [21] phages are the most studied viruses and used for the rapid and efficient development of antigen-specific biomolecules, such as vaccines and antibodies. According to the types of display and the shapes of virions, the distilled antigen-binders can either be isolated from or remain connected with the virions to serve as an efficient recognition interface between the analyte and the signal transducer of a sensor.

**Figure 2.**
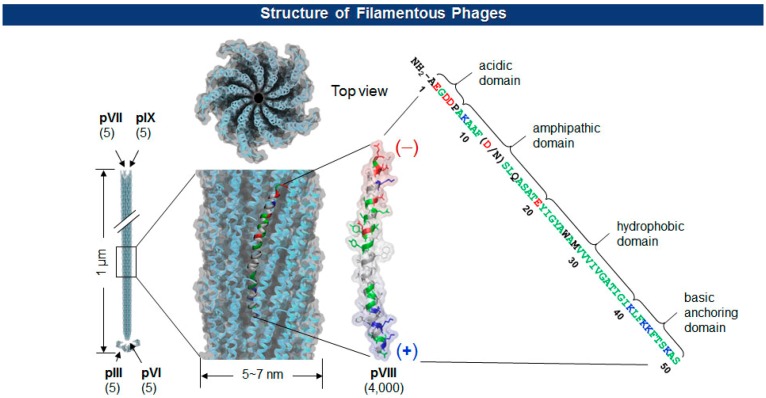
Filamentous phages (Ff class) enclose single-stranded viral DNA with five capsid proteins. Their genomes are >98% identical with interchangeable gene products.

The Ff class of filamentous phage (f1, fd, and M13; ∼1 μm long and ∼7 nm wide) is a non-enveloped and non-lytic virus in which single-stranded DNA (ssDNA) is enclosed by five capsid proteins (pIII, pVI, pVII, pVIII, and pIX). The number of minor coat proteins at each tip of the virion (pIII and pVI are on one end, pVII and pIX are on the other) ranges from three to five copies, while the major coat protein pVIII constitutes 2700∼4000 copies depending upon the length of the viral genes [20] (Figure 2). With pIII in the lead, each of the coat protein has been successfully employed for the display of polypeptides [21–23].

**Figure 3.**
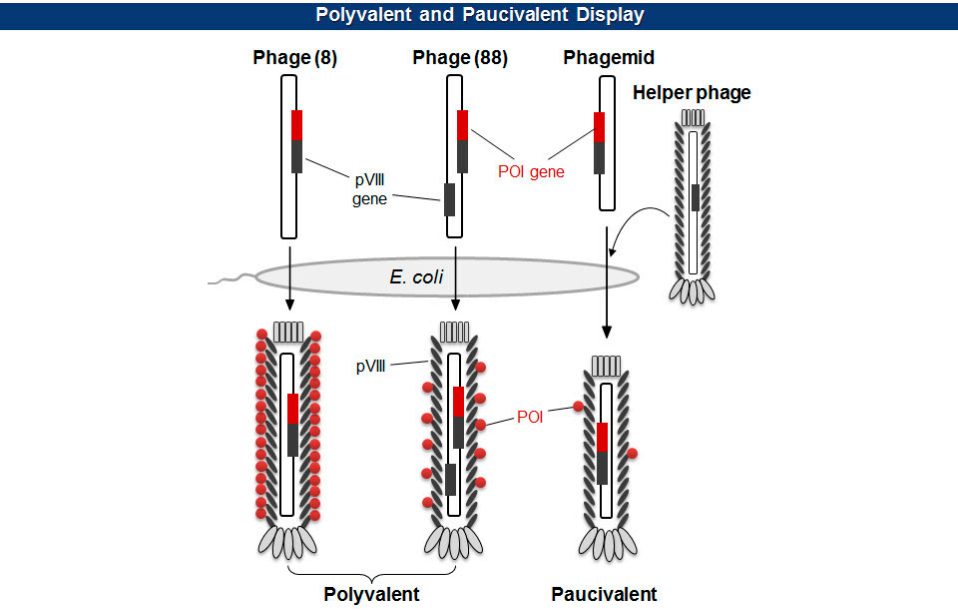
Types of phage display via major capsid protein pVIII as an example. Type 8 uses genomic pVIII as a fusion partner, yielding every pVIII gene product display protein of interest (POI). Type 88 uses additional pVIII as a fusion partner, which gives less amount of POI. Phagemid and helper phage give rise to paucivalent display.

There are two types of phage displays in terms of the copy number of fusion proteins: multivalent (or polyvalent) and paucivalent (or monovalent) [20–23] (Figure 3). The multivalent display has been accomplished using phage vectors and is usually required for the initial screening of scaffold proteins, such as scFv and Fab, which have lower affinity. The paucivalent display is realized in two ways: phagemid systems with “helper” phages and phage vectors with additional recombinant capsid gene, typically, pVIII. A phagemid is a plasmid encoding one of the viral capsid proteins fused to a molecule to be displayed, while a helper phage provides the other viral proteins necessary for the viral assembly [24]. Since phagemids carry filamentous phage replication origin (f1 ori), which is usually compromised in helper phages, the final virion contains mostly the phagemid gene. This enables continuous selections of phagemid gene during several rounds of panning although there are some deviations where helper phages are preferred owing to the expression bias of libraries. Using a single phage vector carrying an additional coat protein resolves the selection bias problem, but the cloning for library generation is rather arduous because of its relatively larger plasmid size and low tolerance to genetic mutations. The paucivalent display system is exploited at later steps of affinity maturation and generally yields higher affinity protein scaffolds [25].

## 3. Multivalent Phage Display

A major coat protein, pVIII, is the best fusion host for the multivalent display of polypeptides because of its natural high copy numbers that provide higher affinity [26]. Of note, multivalent display to cover the surface of a phage virion is becoming of great importance because the surface-engineered functional virions find their positions in hybrid nanomaterials and biomaterials. For example, pVIII-mediated display of polypeptides that bind to specific substrates, such as inorganics or biomolecules, the whole phage particles can be hybridized with those third party materials and become a part of batteries [8,9,27], photocatalyst [28–31], imaging agents [32–35], and biosensors [13]. However, care must be taken in fusing pVIII capsid with foreign proteins because of its low tolerance to the factors that alter the size and structure of pVIII. While most of the short peptides less than 12-mer can be displayed via phage pVIII [36–38], frequent and unpredictable exceptions impede pVIII display for short peptides [39–41], which necessitate vigorous screening of amino acid linkers for fusion. In 1996, Perham and coworkers [37] showed that some large peptides can be displayed better than the shorter ones. By scrutinizing the process rate of signal peptides, they concluded that α-helical structure, beginning about 14th residue from the *N*-terminus of a display peptide, severely hinders the cleavage of signal peptides and thereby hampers the pVIII incorporation to the virion shell [37]. Although rarely, larger proteins such as human growth hormone and streptavidin can be multivalently displayed via pVIII fusion using phagemid-helper phage hybrid systems [42]. In this case, however, different mutation profiles of pVIII itself, selected through directed evolution, should be implemented within pVIII for each protein.

**Figure 4.**
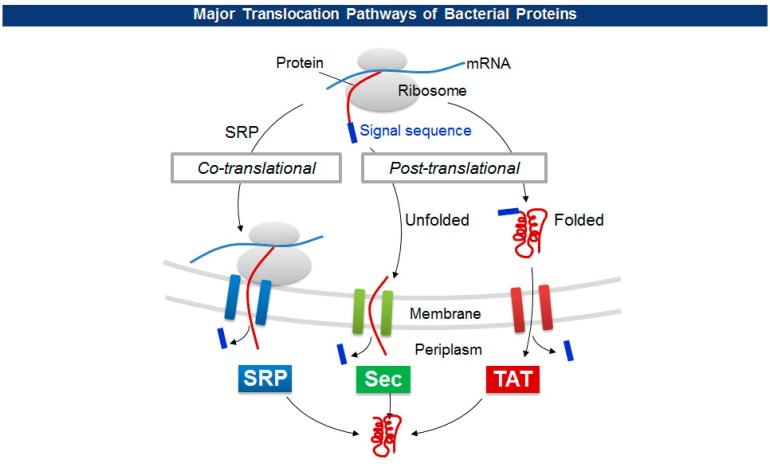
Extracellular proteins initially translocate into periplasm according to the types of signal sequences: SRP, Sec, and TAT-dependent signal sequences.

The size limitations of pVIII fusion proteins may originate from the diameter of a cell membrane export channel composed of viral proteins (pIV and pI/pXI) where *in situ* viral assembly and extrusion from bacteria occur [43]. To overcome the size barrier, researchers have employed a variety of unconventional pVIII display. For one, adapter-directed phage display uses a pair of heterodimeric coiled-coil sequences for each phagemid and helper phage. The phagemid contains Fab molecules flanked by a coiled-coil adapter (GR1), while the helper phage carries the other adapter (GR2) in front of a capsid gene. The level of Fab display, however, stayed below average in spite of a significant higher expression of GR2-pVIII [44]. Working on the other side, Thammawong *et al.* used a different kind of signal peptide that exports a processed protein to the cellular periplasm via the twin-arginine translocation (TAT) pathway [45]. Unlike the conventional Sec-dependent pathway that transport an unfolded target protein to the periplasm during translation, the TAT pathway transports a translated and folded protein [46] (Figure 4). Other signal sequences have been used to explore the effect on the pVIII display on phage virions (Table 1).

In an effort to display even larger cytosolic proteins, the Belcher group at MIT used sortase-mediated ligation to display GFP, a cytosolic protein notorious for its negligible display yield, with 100-fold increase in the copy number (∼100) otherwise unachievable [50]. Subsequently, they utilized sortase for the orthogonal labeling of each capsid protein of M13 phages to generate end-to-end multiphage structures [51]. While this enzyme-mediated surface modification is not compatible with typical phage display of libraries, the methodology itself renders yet another way of surface modification of viruses for the applications to hybrid biomaterials.

## 4. Sensing the Sensitive

**Figure 5.**
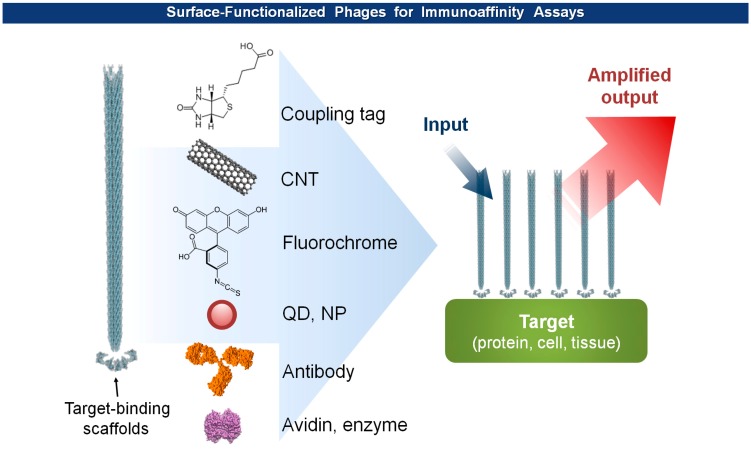
The cylindrical surface of a target-specific phage can be modified with a variety of functional molecules, such as biotin, peptide motifs, optical labels, antibodies, and enzymes to provide augmented and target-specific output signals.

The multiple assembly of coat proteins of viruses provides ample surface-exposed functional groups that aid chemical and enzymatic modifications as well as genetic incorporation of functional scaffolds. In addition, the macromolecular structure renders superior stability in a wide range of pH values and temperatures, and resistance to nucleases and proteases [52,53], a required virtue of a transducer interface. Furthermore, the pH-dependent, thermal, and mechanical stability of the virion particles can be improved either by rational engineering or directed evolution [54,55]. In general, to build an antigen specific biosensor, a target-specific binder is screened and isolated through phage display technology, mass-produced either by synthesis or purification from bacterial cultures, and immobilized on a sensor surface to form a transducer interface [56–58]. In contrast, the basic platform of virus-based biosensors have begun with virions themselves, without isolation of binding scaffolds, that display a target-specific binders. Traditional analytical methods, including mass, optical, electrochemical detection, can be integrated with the virus probes to produce effective biosensors (Figure 5). Since the examples are reviewed in great detail in reference [7], a brief update should suffice to describe this section.

### 4.1. Phages as Molecular Recognition Probes

#### Immunoassays

**Figure 6.**
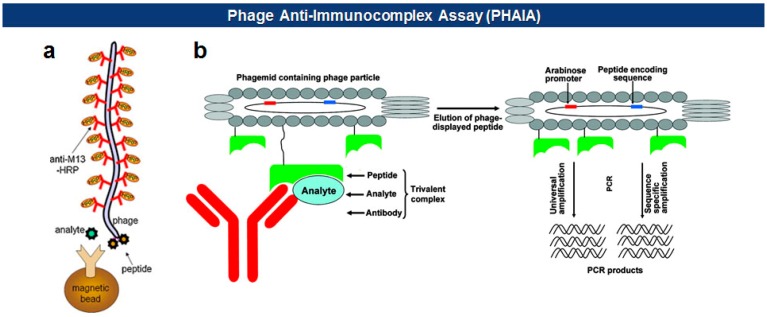
(**a**) The increased surface area due to filamentous phages enhances detection signals using enzyme [61] (Copyright © 2011 Elsevier); (**b**) Analyte-bound phages can be amplified and identified by real-time PCR [63] (Copyright © 2010 ACS).

Phage anti-immunocomplex assay (PHAIA), developed by González-Sapienza and coworkers in 2007 [59,60], uses phage-borne peptides to detect the formation of antibody-antigen immunocomplexes. Initially, a plate coated with an anti-analyte antibody is used to select phages carrying pVIII-fused peptide libraries that bind to the analyte. The selected anti-analyte phage particles then replace detection antibodies in two-site sandwich immunoassays. The long thread-like phages bound to the analyte-antibody immunocomplex can be visualized, with an increased sensitivity due to a larger surface area, by using HRP-conjugated anti-phage antibodies. Using this technique, they successfully detected small molecules, such as molinate [59,61], phenoxybenzoic acid (PBA) [60,62,63], brominated diphenyl ether 47 (BDE 47) [64], and clomazone [65]. Notably, the PHAIA method works well with a magnetic microbead-based immunoassay [62], PCR [63], and magneto-electrochemical immunoassay [61] (Figure 6). Similarly, a landscape phage developed by Petrenko *et al.* in 1996 [39], carrying an analyte binding motif on all copies of the major coat protein pVIII, can replace primary antibodies with an exceptional multivalency in a sandwich immunoassay [66]. Another way of using filamentous phages as antibody surrogates is to chemically couple reporter molecules, such as horseradish peroxidase (HRP) [67], fluorochrome [68], nanoparticles (NPs) [69], carbon nanotubes (CNTs) [35], and quantum dots (QDs) [70], to supersede, or even to skip, a labeling step with conventional secondary antibodies (see Figure 5).

Detection and identification of cancer cells and biomarkers *in vivo* occupy a growing portion of biosensor applications. Since extracellular proteins, especially cell surface receptors, change their population according to the type and physiological status of cells, identification of a cell surface protein can be of great importance in setting the clinical status and therapy of patients. Cell-based phage display panning has proven to be useful for the identification of binders, such as Fab, scFv, or peptide, against cell surface marker proteins [71–74]. The cell-based panning is preferred to a typical plate-based antigen panning in finding true binders because, oftentimes, the marker proteins maintain their intact structures and characters on live cell membranes [75]. The selected cell binding antibodies undergo humanization process to eventually generate therapeutic antibodies that bind cell surface proteins to block cell-cell interactions, thereby elicit antibody dependent cellular cytotoxicity (ADCC) or complement dependent cytotoxicity (CDC), a targeted killing of malicious cells. More efficiently, the whole phage virions displaying target binding scaffolds can be directly used to locate and identify a specific cell, and, more desirably, to deliver various payloads [74,75]. Conversely, if we immobilize the target-specific phage virions on a transducer interface of a certain type of a sensor, we may identify target cells among the mixture of various types of cells. For example, metastatic cells, SW620, were detected using a phage peptide-modified light-addressable potentiometric sensor (LAPS) [76]. The LAPS monitors the changes in photocurrent through an electrolyte-insulator-semiconductor interface induced by an addressable scanning light [77]. The SW620 specific peptides were selected by cell-based panning and then immobilized on a LAPS chip. The captured cells were then sensed by a lock-in amplifier and translated into output voltages. The phage-based LAPS could distinguish metastatic from non-metastatic cells, detecting as low as 100 metastatic cells per ml of blood. To extend the applicability of phage-based probes, Francis and co-workers modified fd bacteriophages to incorporate cage-like xenon-binding molecules (CryA) on pVIII, while the phages are expressing antibodies for epidermal growth factor receptor (EGFR) on pIII minor coat proteins [78]. By doing so, the modified virions can bind to EGFR-positive cancer cells and be detected by hyperpolarized ^129^Xe NMR spectroscopy using chemical exchange saturation transfer (hyperCEST).

### 4.2. Phages as Structural Elements

When the phage virions participate the sensing event, they not only serve as a support for recognition probes, but provides structural dimensions from which a remarkable amplification of signals originate. To that end, filamentous viruses are subjected to surface functionalization either by chemical modification or introducing a short peptide motif to a major coat protein pVIII.

#### 4.2.1. Virus-Polymer Hybrids

**Figure 7.**
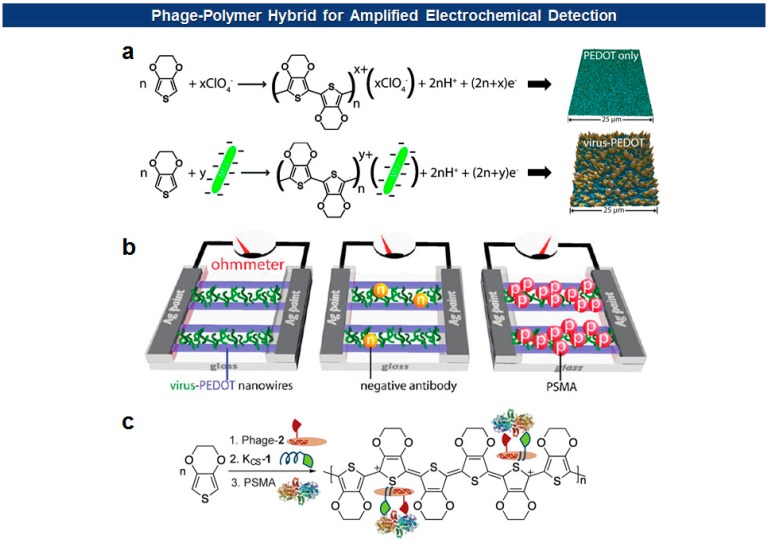
(**a**) EDOT polymerization with and without M13 virus [82]; (**b**) Detection of PSMA as a resistance increase for an array of PSMA-binding virus-PEDOT composite nanowires [49]; (**c**) Phages modified with dual ligands for PSMA can form PEDOT composite to create virus-electrode for the detection of PSMA with a higher sensitivity [83] (Copyright © 2011-2013 ACS).

Frequently, antigen-specific viruses form virus-polymer hybrid composite films or nanowires for the electrochemical detection of the specific antigen. Penner and Weiss have devised a “virus electrode” for the electrochemical detection of analytes [79,80]. More recently, they utilized filamentous phages to form virus-PEDOT (poly(3,4-ethylenedioxythiophene)) biocomposite nanowires and films for the electrochemical detection of cognate antibodies or prostate-specific membrane antigen (PSMA), a prostate cancer biomarker [49,81–83] (Figure 7). They initially identified a PSMA binding motif (SECVEVFQNSCDW) from pVIII-displayed peptide libraries. Then the mixture of phage virions carrying the binders on their surface and EDOT (3,4-ethylenedioxythiophene) monomers was electrodeposited on nanowire electrodes [49]. In this way, PEDOT, a well-known conducting polymer, provides a high degree of malleability, flexibility, and porosity suitable for the biosensing applications, while M13 phage virions offer versatile platforms for molecular recognition.

#### 4.2.2. Virus-NPs Hybrids

Souza *et al.* have shown that the major coat protein pVIII can mediate spontaneous formation of AuNPs at acidic conditions [84]. As expected, the nanoparticular structures exerted optical properties, such as surface enhanced Raman scattering (SERS), in proportional to the amount of phages specific to a certain analyte. Likewise, a bifunctional phage, carrying Au-binding motif (VSGSSPDS) on its pVIII and single-chain antibody fragment (scFv) on pIII, can be optically visualized by adding AuNPs to the phage-analyte immunoaffinity complex [85]. In addition, the noble-metal nanoparticles offer electrochemical way of detection of a nanoparticle-specific analyte. For example, to facilitate the formation of AgNPs in large scale, a tri-methionine (MMM) motif was tagged at every N-terminus of pVIII of fd virus followed by deposition on an electrode, enabling electrochemical detection of glucose that specifically interact with AgNPs generated on virus particles [86,87].

**Figure 8.**
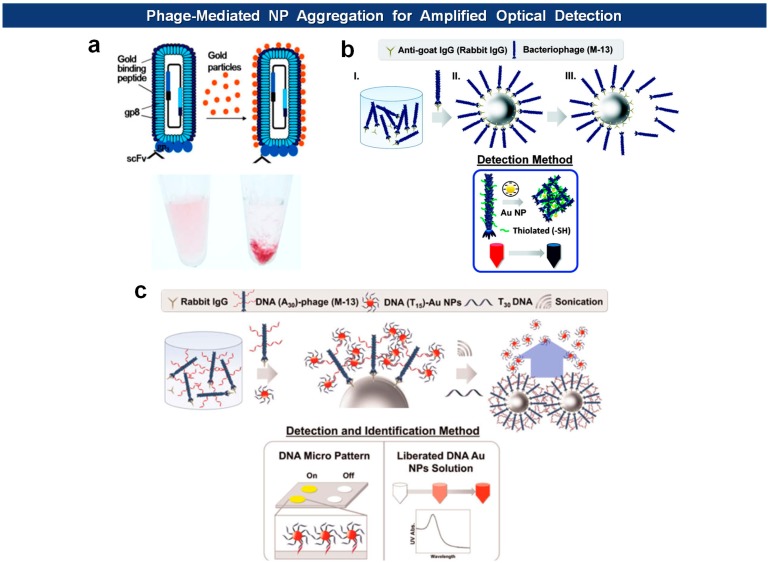
Antigen-specific phages displaying Au-binding motifs (**a**); thiol groups (**b**); or ssDNA (**c**) on their surfaces aggregate in the presence of Au particles [85,88] or recruit ssDNA-AuNPs [89] for easy detection (Copyright © 2014 Elsevier; 2011, 2012 ACS).

Other recent applications include amplified optical protein detection where the macromolecular structure of filamentous virions aids an optical detection of proteins (Figure 8). For example, Cha and coworkers employed antigen-specific M13 bacteriophages of which surfaces are modified with thiol groups [88]. After capturing antigens with magnetic microspheres, the phages are mixed with antigen-loaded microspheres, washed, eluted from the beads, and then reacted with Au nanoparticles (AuNPs) to form aggregates. The degree of aggregation is optimally measured to give a quantitative readout. In another example, they modified the N-terminus of pVIII capsids with phage virions with amine-terminated poly(A_30_) [89]. Again, the phages are captured by antigen-loaded microspheres, and this time reacted with AuNPs loaded with poly(T_15_) oligonulceotides. The bound AuNPs, released by poly(T_30_) and sonication, are directly analyzed by UV-vis spectrometry, and the DNA sequence was identified with a DNA microarray. Likewise, horseradish peroxidase (HRP), an enzyme catalyzing the conversion of chromogenic substrates into colored products, can be linked with DNA instead of AuNPs [90]. Using this “DNAzyme” they lowered the experimental detection limit down to 10 fmol (50 pM).

#### 4.2.3. Virus-Microspheres

**Figure 9.**
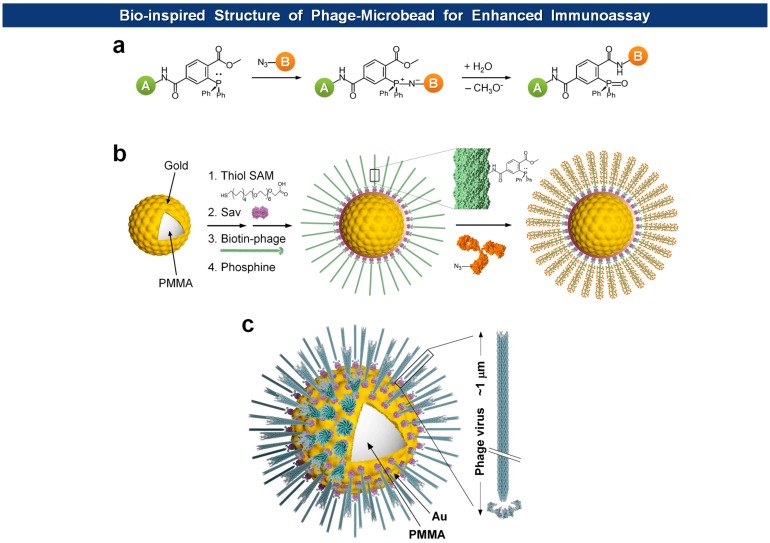
Schematics of Staudinger ligation (**a**) used in the fabrication of virus-Au microspheres (**b**); (**c**) Schematic representation of a bio-inspired virus-Au microsphere [91] (Copyright © 2013 WILEY-VCH).

Microsphere-based suspension arrays are emerging as next-generation sensing formats for multiplexed detection of biomolecules within a limited sample volume. Filamentous phages have been immobilized on magnetic microspheres for PHAIA [62], serum protein analysis [92], and affinity separation [93]. Recently, Jeon *et al.* immobilized fd virions on Au-coated microspheres with orientation-controlled manner, providing exceptionally large surface to volume ratio for the immobilization of capture antibodies in high density [91] (Figure 9). They envisioned that the dimension of microspheres and the morphology of filamentous viruses should offer a unique opportunity for building of biomimetic architectures. Since most cells have evolved to enhance cell-to-cell or cell-to-ligand interactions, they usually manifest thread-like tentacles such as microvilli [94], filopodia [95], pili and fimbriae [96,97]. Furthermore, in biorecognition processes, such as ligand-receptor interactions, longer and flexible tethers and polyvalency have proven to be beneficial [98–101]. The final structure of virus-tethered microspheres is reminiscent of cells, such as macrophages, leukocytes, and some epithelial cells, that have surface threads for the enhanced cellular movement, adhesion, and endocytosis. In addition, to prevent pandemic nonspecific adsorption observed in neat serum samples using conventional polystyrene or poly(methyl methacrylate) (PMMA) microbeads, they covered the bead with a thin Au layer on which a self-assembled monolayer (SAM) of carboxyl-terminated polyethylene glycol (PEG) alkanethiolates is formed. In this way, the high density of carboxyl groups and PEG moieties provides efficient immobilization of functional molecules and prevention of nonspecific adsorption, respectively. Indeed, when compared to normal microspheres, the bio-inspired and Au protected virus-microspheres exhibited enhanced detection of cardiac marker cTnI, as low as 20 pg/mL in neat blood serum.

**Figure 10.**
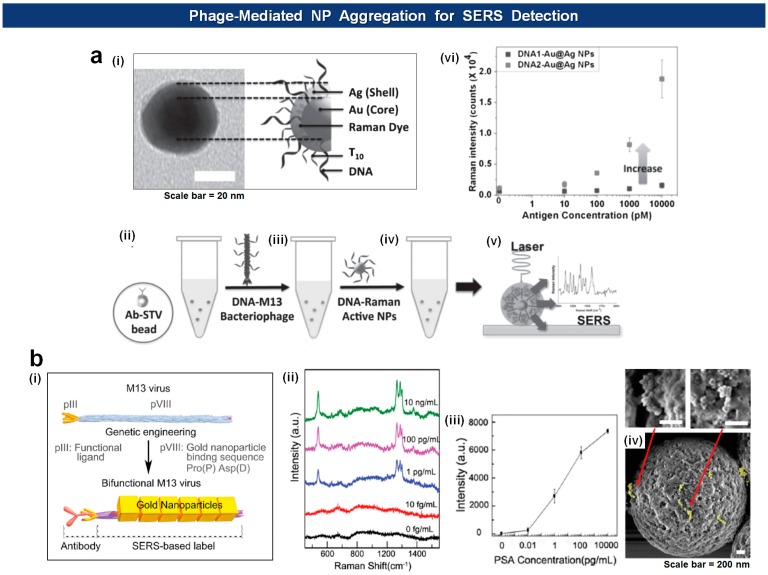
Phage-NPs for the SERS-based detection of analytes. (**a**) DNA-Cy3-Au@Ag (i) used in the SERS generation of bead-captured M13 virus. Panel (ii)-(vi) show working principle and enhanced detection profiles [102]; (**b**) Fabrication of phage-AuNCs hybrids (i) for the detection of PSA (ii-iii) by capturing the virus probe using magnetic beads (iv) [103] (Copyright © 2014 WILEY-VCH).

Recently, the Cha group has extended the application of the DNA-loaded and the DNA-loaded M13 phages by merging them onto silica microbeads and thereby elicit quantitative SERS activity depending upon the amount of antigens [102]. In this setup, the antibody-loaded beads capture antigens that are recognized by the antigen-specific phages analogously to sandwich-type immunoassays. The NPs are then coupled with phages via complimentary DNA interaction. This way they could achieve SERS enhancement factor up to ∼10^6^ and detect 10 pM (5 fmol) of model antigen (Figure 10a). A similar approach was reported by Lee *et al.* [103] who used bifunctional M13 virus on which Au nanocubes (AuNCs) were attached through a AuNCs-binding motifs (pro-asp) on pVIII major coat proteins. The virus-based SERS nanoprobes, debbed V-probes, were captured by magnetic beads after incubation with PSA antigens, followed by SERS analysis of the beads, yielding limit of detection 1 fg/mL (Figure 10b).

#### 4.2.4. Virus Self-Templating Assembly

**Figure 11.**
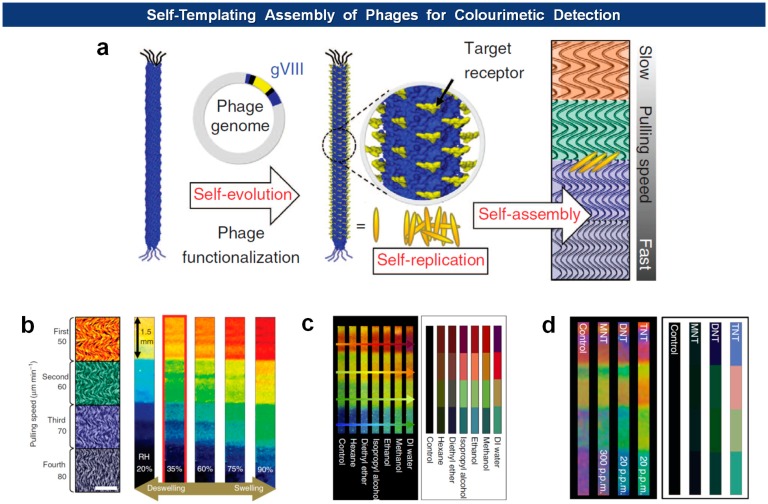
Phage Litmus as colourimetric sensors. (**a**) Schematics for the generation of self-templating assembly of functionalized phages onto a substrate with varying deposition speed. Colour changes of the Phage Litmus due to humidity (**b**); VOCs (**c**); and TNT (**d**) [105] (Copyright © 2014 Macmillan Publishers Ltd.).

Previously, Lee and colleagues demonstrated that the M13 phages could undergo self-templating assembly to form supramolecular film structures [104]. The liquid crystal phase of the assembled phages can be regulated by adjusting the phage concentration, pulling speed, ionic concentration, phage surface chemistry, and substrate surface properties. Recently, they adroitly adopted the tunable phage-based structures that exhibit unique optical properties into colourimetric sensors, termed Phage Litmus [105]. They generated multi-coloured matrices composed of quasi-ordered phage bundles by controlling the phage deposition speed, that is, by controlling the pulling speed of a solid substrate that had been submerged in a phage solution. The colours of the phage matrix bands change due to transition of the bundled structure of the phages. Thus, depending upon the phage libraries selected by directed evolution, the Phage Litmus could sense humidity, volatile organic compounds (VOCs), and trinitrotoluene (TNT) (Figure 11).

## 5. Conclusions and Outlook

The unique biological, mechanical, and geometrical characters of viruses offer a cornucopia of opportunities for chemical and biological sensors. The versatile mode of protein and peptide display of filamentous phages enables site-specific immobilization of the virions displaying multiple copies of ligands or functional molecules. Furthermore, the ongoing development of virus-based hybrid nanomaterials ever enriches the content of virus-based biosensors.

The phage display technology itself, however, still has room for improvement in terms of pVIII-based multivalent display of some cytosolic or large proteins. Conventional applications may do without such a multivalent display because a paucivalent display of libraries, via other than pVIII, have almost always yielded reasonable selection of target binding scaffolds. In contrast, with the advent of virus-based hybrid materials, precise control of multivalent and paucivalent display, either by engineering the membrane channels or signal sequences, would be necessary for the extended application of virions on the sensor transducers.

Thread-like viruses provide yet another dimension of sensor application, a bio-inspired architecture that can enhance the interactions between the sensor and its target, be it ever peptide, protein, or a cell. Like many other sensor applications that involve surface modification with functional molecules [106,107], fabrication of a specific biomimetic structure using virions usually require an orientation-controlled immobilization of phages as well as an orientation-controlled conjugation of functional molecules on phages [108]. Active participation of well-established bioorthogonal and chemoselective reactions, such as azide-alkyne click chemistry, Streptavidin-biotin binding, Staudinger ligation, and Diels-Alder reactions [109,110], together with genetic engineering for the incorporation of unnatural amino acids [111–113], will further raise the degree of versatility, and thus expand the methodology, of the virus-based sensors in the near future.

## Figures and Tables

**Table 1.** Representative signal sequences used for pVIII-mediated phage display.

**Display**	**Phagemid**	***Promoter* Signal Peptide**	**Note**	**Ref.**
Human growth hormone Streptavidin	pS657a/pS1607	*tac*	pVIII variant (different residues at positions 11–20)	[42]
		MalE (Sec) ^1^		

C147Ex	pTat8	*lac*	Effect of twin-arginine signal peptide	[45]
	pComb8	TorA (TAT) ^2^	Effect of IPTG and infection timing	[47]
		PelB (Sec) ^3^		

EX_2_CXPGXTXKX_2_CNTC XCX_3_GX_3_ACTXKXCPX_2_	Tag-pGP8: pS1607 derivative	*lac*	FLAG tagged Pacifastin-related inhibitor	[48]
		PhoA (Sec) ^4^		

SECVEVFQNSCDW	pM1165a derivative	*tac*	Prostate specific membrane antigen (PSMA) binding peptide	[49]
		DsbA (SRP) ^5^		

1 MalE: maltose binding protein (MBP) signal peptide.

2 TorA: trimethylamine *N*-oxide reductase.

3 PelB: pectate lyase B.

4 PhoA: bacterial alkaline phosphatase.

5 DsbA: periplasmic disulfide bond oxidoreductase.

## References

[1] Nagel B., Dellweg H., Gierasch L.M. (1992). Glossary for chemists of terms used in biotechnology—(IUPAC Recommendations 1992). Pure Appl. Chem..

[2] Zacher A.N., Stock C.A., Golden J.W., Smith G.P. (1980). A new filamentous phage cloning vector: Fd-tet. Gene.

[3] Nelson F.K., Friedman S.M., Smith G.P. (1981). Filamentous phage DNA cloning vectors—a non-infective mutant with a non-polar deletion in gene-III. Virology.

[4] Smith G.P. (1985). Filamentous fusion phage—novel expression vectors that display cloned antigens on the virion surface. Science.

[5] Whaley S.R., English D.S., Hu E.L., Barbara P.F., Belcher A.M. (2000). Selection of peptides with semiconductor binding specificity for directed nanocrystal assembly. Nature.

[6] Lee S.W., Mao C., Flynn C.E., Belcher A.M. (2002). Ordering of quantum dots using genetically engineered viruses. Science.

[7] Mao C., Solis D.J., Reiss B.D., Kottmann S.T., Sweeney R.Y., Hayhurst A., Georgiou G., Iverson B., Belcher A.M. (2004). Virus-based toolkit for the directed synthesis of magnetic and semiconducting nanowires. Science.

[8] Nam K.T., Kim D.W., Yoo P.J., Chiang C.Y., Meethong N., Hammond P.T., Chiang Y.M., Belcher A.M. (2006). Virus-enabled synthesis and assembly of nanowires for lithium ion battery electrodes. Science.

[9] Lee Y.J., Yi H., Kim W.J., Kang K., Yun D.S., Strano M.S., Ceder G., Belcher A.M. (2009). Fabricating genetically engineered high-power lithium-ion batteries using multiple virus genes. Science.

[10] Fan X.Z., Pomerantseva E., Gnerlich M., Brown A., Gerasopoulos K., McCarthy M., Culver J., Ghodssi R. (2013). Tobacco mosaic virus: A biological building block for micro/nano/bio systems. J. Vac. Sci. Technol. A.

[11] Reiss B.D., Mao C.B., Solis D.J., Ryan K.S., Thomson T., Belcher A.M. (2004). Biological routes to metal alloy ferromagnetic nanostructures. Nano Lett..

[12] Huang Y., Chiang C.Y., Lee S.K., Gao Y., Hu E.L., De Yoreo J., Belcher A.M. (2005). Programmable assembly of nanoarchitectures using genetically engineered viruses. Nano Lett..

[13] Mao C.B., Liu A.H., Cao B.R. (2009). Virus-based chemical and biological sensing. Angew. Chem. Int. Ed..

[14] Park J.S., Cho M.K., Lee E.J., Ahn K.Y., Lee K.E., Jung J.H., Cho Y., Han S.S., Kim Y.K., Lee J. (2009). A highly sensitive and selective diagnostic assay based on virus nanoparticles. Nat. Nanotechonol..

[15] Lee J.W., Song J., Hwang M.P., Lee K.H. (2013). Nanoscale bacteriophage biosensors beyond phage display. Int. J. Nanomed..

[16] Singh A., Arutyunov D., Szymanski C.M., Evoy S. (2012). Bacteriophage based probes for pathogen detection. Analyst..

[17] Singh A., Poshtiban S., Evoy S. (2013). Recent advances in bacteriophage based biosensors for food-borne pathogen detection. Sensors.

[18] Tawil N., Sacher E., Mandeville R., Meunier M. (2014). Bacteriophages: Biosensing tools for multi-drug resistant pathogens. Analyst..

[19] Larman H.B., Zhao Z.M., Laserson U., Li M.Z., Ciccia A., Gakidis M.A. M., Church G.M., Kesari S., LeProust E.M., Solimini N.L., Elledge S.J. (2011). Autoantigen discovery with a synthetic human peptidome. Nat. Biotechnol..

[20] Smith G.P., Petrenko V.A. (1997). Phage display. Chem. Rev..

[21] Kehoe J.W., Kay B.K. (2005). Filamentous phage display in the new millennium. Chem. Rev..

[22] Hoess R.H. (2001). Protein design and phage display. Chem. Rev..

[23] Loset G.A., Sandlie I. (2012). Next generation phage display by use of pVII and pIX as display scaffolds. Methods.

[24] Qi H., Lu H.Q., Qiu H.J., Petrenko V., Liu A.H. (2012). Phagemid vectors for phage display: properties, characteristics and construction. J. Mol. Biol..

[25] O'Connell D., Becerril B., Roy-Burman A., Daws M., Marks J.D. (2002). Phage *versus* phagemid libraries for generation of human monoclonal antibodies. J. Mol. Biol..

[26] Knez K., Noppe W., Geukens N., Janssen K.P. F., Spasic D., Heyligen J., Vriens K., Thevissen K., Cammue B.P. A., Petrenko V., Ulens C., Deckmyn H., Lammertyn J. (2013). Affinity comparison of p3 and p8 peptide displaying bacteriophages using surface plasmon resonance. Anal. Chem..

[27] Lee Y.J., Lee Y., Oh D., Chen T., Ceder G., Belcher A.M. (2010). Biologically activated noble metal alloys at the nanoscale: for lithium ion battery anodes. Nano Lett..

[28] Nam Y.S., Magyar A.P., Lee D., Kim J.W., Yun D.S., Park H., Pollom T.S., Weitz D.A., Belcher A.M. (2010). Biologically templated photocatalytic nanostructures for sustained light-driven water oxidation. Nat. Nanotechonol..

[29] Dang X., Yi H., Ham M.H., Qi J., Yun D.S., Ladewski R., Strano M.S., Hammond P.T., Belcher A.M. (2011). Virus-templated self-assembled single-walled carbon nanotubes for highly efficient electron collection in photovoltaic devices. Nat. Nanotechonol..

[30] Nuraje N., Dang X.N., Qi J.F., Allen M.A., Lei Y., Belcher A.M. (2012). Biotemplated synthesis of perovskite nanomaterials for solar energy conversion. Adv. Mater..

[31] Nam Y.S., Shin T., Park H., Magyar A.P., Choi K., Fantner G., Nelson K.A., Belcher A.M. (2010). Virus-templated assembly of porphyrins into light-harvesting nanoantennae. J. Am. Chem. Soc..

[32] Deutscher S.L. (2010). Phage display in molecular imaging and diagnosis of cancer. Chem. Rev..

[33] Carrico Z.M., Farkas M.E., Zhou Y., Hsiao S.C., Marks J.D., Chokhawala H., Clark D.S., Francis M.B. (2012). N-Terminal labeling of filamentous phage to create cancer marker imaging agents. ACS Nano.

[34] Ghosh D., Kohli A.G., Moser F., Endy D., Belcher A.M. (2012). Refactored M13 bacteriophage as a platform for tumor cell imaging and drug delivery. ACS Synth. Biol..

[35] Yi H.J., Ghosh D., Ham M.H., Qi J.F., Barone P.W., Strano M.S., Belcher A.M. (2012). M13 phage-functionalized single-walled carbon nanotubes as nanoprobes for second near-infrared window fluorescence imaging of targeted tumors. Nano Lett..

[36] Iannolo G., Minenkova O., Petruzzelli R., Cesareni G. (1995). Modifying filamentous phage capsid—limits in the size of the major capsid protein. J. Mol. Biol..

[37] Malik P., Tarry T.D., Gowda L.R., Langara A., Petukhov S.A., Symmons M.F., Welsh L.C., Marvin D.A., Perham R.N. (1996). Role of capsid structure and membrane protein processing in determining the size and copy number of peptides displayed on the major coat protein of filamentous bacteriophage. J. Mol. Biol..

[38] Nam K.T., Lee Y.J., Krauland E.M., Kottmann S.T., Belcher A.M. (2008). Peptide-mediated reduction of silver ions on engineered biological scaffolds. ACS Nano.

[39] Petrenko V.A., Smith G.P., Gong X., Quinn T. (1996). A library of organic landscapes on filamentous phage. Protein Eng..

[40] Petrenko V.A., Smith G.P., Mazooji M.M., Quinn T. (2002). Alpha-helically constrained phage display library. Protein Eng..

[41] Merzlyak A., Lee S.W. (2009). Engineering phage materials with desired peptide display: rational design sustained through natural selection. Bioconj. Chem..

[42] Sidhu S.S., Weiss G.A., Wells J.A. (2000). High copy display of large proteins on phage for functional selections. J. Mol. Biol..

[43] Opalka N., Beckmann R., Boisset N., Simon M.N., Russel M., Darst S.A. (2003). Structure of the filamentous phage pIV multimer by cryo-electron microscopy. J. Mol. Biol..

[44] Wang K.C., Wang X.W., Zhong P.Y., Luo P.P. (2010). Adapter-directed display: A modular design for shuttling display on phage surfaces. J. Mol. Biol..

[45] Thammawong P., Kasinrerk W., Turner R.J., Tayapiwatana C. (2006). Twin-arginine signal peptide attributes effective display of CD147 to filamentous phage. Appl. Microbiol. Biotechnol..

[46] Mergulhao F.J.M., Summers D.K., Monteiro G.A. (2005). Recombinant protein secretion in *Escherichia coli*. Biotechnol. Adv..

[47] Intasai N., Arooncharus P., Kasinrerk W., Tayapiwatana C. (2003). Construction of high-density display of CD147 ectodomain on VCSM13 phage via gpVIII: Effects of temperature, IPTG, and helper phage infection-period. Protein Expr. Purif..

[48] Szenthe B., Patthy A., Gaspari Z., Kekesi A.K., Graf L., Pal G. (2007). When the surface tells what lies beneath: combinatorial phage-display mutagenesis reveals complex networks of surface-core interactions in the pacifastin protease inhibitor family. J. Mol. Biol..

[49] Arter J.A., Diaz J.E., Donavan K.C., Yuan T., Penner R.M., Weiss G.A. (2012). Virus-polymer hybrid nanowires tailored to detect prostate-specific membrane antigen. Anal. Chem..

[50] Hess G.T., Cragnolini J.J., Popp M.W., Allen M.A., Dougan S.K., Spooner E., Ploegh H.L., Belcher A.M., Guimaraes C.P. (2012). M13 bacteriophage display framework that allows sortase-mediated modification of surface-accessible phage proteins. Bioconj. Chem..

[51] Hess G.T., Guimaraes C.P., Spooner E., Ploegh H.L., Belcher A.M. (2013). Orthogonal labeling of M13 minor capsid proteins with DNA to self-assemble end-to-end multiphage structures. ACS Synth. Biol..

[52] Barbas C.F., Burton D.R., Scott J.K., Silverman G.J. (2004). Phage Display: A Laboratory Manual.

[53] Brigati J.R., Petrenko V.A. (2005). Thermostability of landscape phage probes. Anal. Bioanal. Chem..

[54] Mateu M.G. (2011). Virus engineering: Functionalization and stabilization. Protein Eng. Des. Sel..

[55] Nasir S.F., Jaworski J. (2014). Assessing the stability of assembled filamentous phage coat protein P8. Supramol. Chem..

[56] Wu J., Cropek D.M., West A.C., Banta S. (2010). Development of a troponin I biosensor using a peptide obtained through phage display. Anal. Chem..

[57] Wu J., Park J.P., Dooley K., Cropek D.M., West A.C., Banta S. (2011). Rapid development of new protein biosensors utilizing peptides obtained via phage display. PLoS One.

[58] Rangnoi K., Jaruseranee N., O'Kennedy R., Pansri P., Yamabhai M. (2011). One-step detection of aflatoxin-B-1 using scFv-alkaline phosphatase-fusion selected from human phage display antibody library. Mol. Biotechnol..

[59] Gonzalez-Techera A., Vanrell L., Last J.A., Hammock B.D., Gonzalez-Sapienza G. (2007). Phage anti-immune complex assay: General strategy for noncompetitive immunodetection of small molecules. Anal. Chem..

[60] Gonzalez-Techera A., Kim H.J., Gee S.J., Last J.A., Hammock B.D., Gonzalez-Sapienza G. (2007). Polyclonal antibody-based noncompetitive immunoassay for small analytes developed with short peptide loops isolated from phage libraries. Anal. Chem..

[61] Arevalo F.J., Gonzalez-Techera A., Zon M.A., Gonzalez-Sapienza G., Fernandez H. (2012). Ultra-sensitive electrochemical immunosensor using analyte peptidomimetics selected from phage display peptide libraries. Biosens. Bioelectron..

[62] Kim H.J., Ahn K.C., Gonzalez-Techera A., Gonzalez-Sapienza G.G., Gee S.J., Hammock B.D. (2009). Magnetic bead-based phage anti-immunocomplex assay (PHAIA) for the detection of the urinary biomarker 3-phenoxybenzoic acid to assess human exposure to pyrethroid insecticides. Anal. Biochem..

[63] Kim H.J., McCoy M., Gee S.J., Gonzalez-Sapienza G.G., Hammock B.D. (2011). Noncompetitive phage anti-immunocomplex real-time polymerase chain reaction for sensitive detection of small molecules. Anal. Chem..

[64] Kim H.J., Rossotti M.A., Ahn K.C., Gonzalez-Sapienza G.G., Gee S.J., Musker R., Hammock B.D. (2010). Development of a noncompetitive phage anti-immunocomplex assay for brominated diphenyl ether 47. Anal. Biochem..

[65] Rossotti M.A., Carlomagno M., Gonzalez-Techera A., Hammock B.D., Last J., Gonzalez-Sapienza G. (2010). Phage anti-immunocomplex assay for clomazone: Two-site recognition increasing assay specificity and facilitating adaptation into an on-site format. Anal. Chem..

[66] Lang Q., Wang F., Yin L., Liu M., Petrenko V.A., Liu A. (2014). Specific probe selection from landscape phage display library and its application in enzyme-linked immunosorbent assay of free prostate-specific antigen. Anal. Chem..

[67] Adhikari M., Dhamane S., Hagstrom A.E. V., Garvey G., Chen W.H., Kourentzi K., Strych U., Willson R.C. (2013). Functionalized viral nanoparticles as ultrasensitive reporters in lateral-flow assays. Analyst..

[68] Kelly K.A., Waterman P., Weissleder R. (2006). *In vivo* imaging of molecularly targeted phage. Neoplasia.

[69] Ghosh D., Lee Y., Thomas S., Kohli A.G., Yun D.S., Belcher A.M., Kelly K.A. (2012). M13-templated magnetic nanoparticles for targeted *in vivo* imaging of prostate cancer. Nat. Nanotechonol..

[70] Zhao W.X., Jin L., Yuan H., Tan Z.Y., Zhou C.H., Li L.S., Ma L. (2013). Targeting human embryonic stem cells with quantum dot-conjugated phages. Sci. Rep..

[71] Andersen P.S., Stryhn A., Hansen B.E., Fugger L., Engberg J., Buus S. (1996). A recombinant antibody with the antigen-specific, major histocompatibility complex-restricted specificity of T cells. Proc. Natl. Acad. Sci. USA.

[72] Barry M.A., Dower W.J., Johnston S.A. (1996). Toward cell-targeting gene therapy vectors: selection of cell-binding peptides from random peptide-presenting phage libraries. Nat. Med..

[73] Jayanna P.K., Bedi D., Deinnocentes P., Bird R.C., Petrenko V.A. (2010). Landscape phage ligands for PC3 prostate carcinoma cells. Protein Eng. Des. Sel..

[74] Gray B.P., Brown K.C. (2014). Combinatorial peptide libraries: Mining for cell-binding peptides. Chem. Rev..

[75] Zhou Y., Zhao L.Q., Marks J.D. (2012). Selection and characterization of cell binding and internalizing phage antibodies. Arch. Biochem. Biophys..

[76] Zhang H.K., Li X., Bai Y.P., Niu R.F., Jia Y.F., Zhang C.Z., Zhang L., Feng X.Z., Cao Y.J. (2009). Metastatic cell detection using a phage-peptide-modified light-addressable potentiometric sensor. Biotechnol. Appl. Biochem..

[77] Hafeman D.G., Parce J.W., Mcconnell H.M. (1988). Light-addressable potentiometric sensor for biochemical systems. Science.

[78] Palaniappan K.K., Ramirez R.M., Bajaj V.S., Wemmer D.E., Pines A., Francis M.B. (2013). Molecular imaging of cancer cells using a bacteriophage-based ^129^Xe NMR biosensor. Angew. Chem. Int. Ed..

[79] Yang L.M.C., Tam P.Y., Murray B.J., McIntire T.M., Overstreet C.M., Weiss G.A., Penner R.M. (2006). Virus electrodes for universal biodetection. Anal. Chem..

[80] Yang L.M.C., Diaz J.E., McIntire T.M., Weiss G.A., Penner R.M. (2008). Covalent virus layer for mass-based biosensing. Anal. Chem..

[81] Arter J.A., Taggart D.K., McIntire T.M., Penner R.M., Weiss G.A. (2010). Virus-PEDOT nanowires for biosensing. Nano Lett..

[82] Donavan K.C., Arter J.A., Pilolli R., Cioffi N., Weiss G.A., Penner R.M. (2011). Virus-poly(3,4-ethylenedioxythiophene) composite films for impedance-based biosensing. Anal. Chem..

[83] Mohan K., Donavan K.C., Arter J.A., Penner R.M., Weiss G.A. (2013). Sub-nanomolar detection of prostate-specific membrane antigen in synthetic urine by synergistic, dual-ligand phage. J. Am. Chem. Soc..

[84] Souza G.R., Christianson D.R., Staquicini F.I., Ozawa M.G., Snyder E.Y., Sidman R.L., Miller J.H., Arap W., Pasqualini R. (2006). Networks of gold nanoparticles and bacteriophage as biological sensors and cell-targeting agents. Proc. Natl. Acad. Sci. USA.

[85] Guo Y.C., Liang X.S., Zhou Y.F., Zhang Z.P., Wei H.P., Men D., Luo M., Zhang X.E. (2010). Construction of bifunctional phage display for biological analysis and immunoassay. Anal. Biochem..

[86] Kang Y.R., Park E.J., Kim J.H., Min N.K., Kim S.W. (2010). Development of bio-nanowire networks using phage-enabled assembly for biological sensor application. Talanta.

[87] Kang Y.R., Hwang K.H., Kim J.H., Nam C.H., Kim S.W. (2010). Disposable amperometric biosensor based on nanostructured bacteriophages for glucose detection. Meas. Sci. Technol..

[88] Lee J.H., Cha J.N. (2011). Amplified protein detection through visible plasmon shifts in gold nanocrystal solutions from bacteriophage platforms. Anal. Chem..

[89] Lee J.H., Domaille D.W., Cha J.N. (2012). Amplified protein detection and identification through DNA-conjugated M13 bacteriophage. ACS Nano.

[90] Domaille D.W., Lee J.H., Cha J.N. (2013). High density DNA loading on the M13 bacteriophage provides access to colorimetric and fluorescent protein microarray biosensors. Chem. Commun..

[91] Jeon C.S., Hwang I., Chung T.D. (2013). Virus-tethered magnetic gold microspheres with biomimetic architectures for enhanced immunoassays. Adv. Funct. Mater..

[92] Zhu G.J., Zhao P., Deng N., Tao D.Y., Sun L.L., Liang Z., Zhang L.H., Zhang Y.K. (2012). Single chain variable fragment displaying M13 phage library functionalized magnetic microsphere-based protein equalizer for human serum protein analysis. Anal. Chem..

[93] Muzard J., Platt M., Lee G.U. (2012). M13 bacteriophage-activated superparamagnetic beads for affinity separation. Small.

[94] Greicius G., Westerberg L., Davey E.J., Buentke E., Scheynius A., Thyberg J., Severinson E. (2004). Microvilli structures on B lymphocytes: inducible functional domains?. Int. Immunol..

[95] Bear M.F., Connors B.W., Paradiso M.A. (2007). Neuroscience: Exploring the Brain.

[96] Connell H., Agace W., Klemm P., Schembri M., Marild S., Svanborg C. (1996). Type 1 fimbrial expression enhances *Escherichia coli* virulence for the urinary tract. Proc. Natl. Acad. Sci. USA.

[97] Lederberg J., Tatum E.L. (1946). Gene recombination in. Escherichia coli. Nature.

[98] Jeppesen C., Wong J.Y., Kuhl T.L., Israelachvili J.N., Mullah N., Zalipsky S., Marques C.M. (2001). Impact of polymer tether length on multiple ligand-receptor bond formation. Science.

[99] Collins B.E., Paulson J.C. (2004). Cell surface biology mediated by low affinity multivalent protein-glycan interactions. Curr. Opin. Chem. Biol..

[100] Wang S.H., Dormidontova E.E. (2011). Nanoparticle targeting using multivalent ligands: computer modeling. Soft Matter..

[101] Reeves D., Cheveralls K., Kondev J. (2011). Regulation of biochemical reaction rates by flexible tethers. Phys. Rev. E.

[102] Lee J.H., Xu P.F., Domaille D.W., Choi C., Jin S., Cha J.N. (2014). M13 bacteriophage as materials for amplified surface enhanced Raman scattering protein sensing. Adv. Funct. Mater..

[103] Lee H.E., Lee H.K., Chang H., Ahn H.Y., Erdene N., Lee H.Y., Lee Y.S., Jeong D.H., Chung J., Nam K.T. (2014). Virus templated gold nanocube chain for SERS nanoprobe. Small.

[104] Chung W.J., Oh J.W., Kwak K., Lee B.Y., Meyer J., Wang E., Hexemer A., Lee S.W. (2011). Biomimetic self-templating supramolecular structures. Nature.

[105] Oh J.W., Chung W.J., Heo K., Jin H.E., Lee B.Y., Wang E., Zueger C., Wong W., Meyer J., Kim C. (2014). Biomimetic virus-based colourimetric sensors. Nat. Commun..

[106] Algar W.R., Prasuhn D.E., Stewart M.H., Jennings T.L., Blanco-Canosa J.B., Dawson P.E., Medintz I.L. (2011). The controlled display of biomolecules on nanoparticles: A challenge suited to bioorthogonal chemistry. Bioconj. Chem..

[107] Sapsford K.E., Algar W.R., Berti L., Gemmill K.B., Casey B.J., Oh E., Stewart M.H., Medintz I.L. (2013). Functionalizing nanoparticles with biological molecules: developing chemistries that facilitate nanotechnology. Chem. Rev..

[108] Smith M.T., Hawes A.K., Bundy B.C. (2013). Reengineering viruses and virus-like particles through chemical functionalization strategies. Curr. Opin. Biotechnol..

[109] Van Berkel S.S., van Eldijk M.B., van Hest J.C.M. (2011). Staudinger ligation as a method for bioconjugation. Angew. Chem. Int. Ed..

[110] Patterson D.M., Nazarova L.A., Prescher J.A. (2014). Finding the right (bioorthogonal) chemistry. ACS Chem. Biol..

[111] Davis L., Chin J.W. (2012). Designer proteins: Applications of genetic code expansion in cell biology. Nat. Rev. Mol. Cell Biol..

[112] Lang K., Chin J.W. (2014). Cellular incorporation of unnatural amino acids and bioorthogonal labeling of proteins. Chem. Rev..

[113] Chin J.W. (2014). Expanding and reprogramming the genetic code of cells and animals. Annu. Rev. Biochem..

